# p190A inactivating mutations cause aberrant RhoA activation and promote malignant transformation via the Hippo-YAP pathway in endometrial cancer

**DOI:** 10.1038/s41392-020-0170-6

**Published:** 2020-05-27

**Authors:** Xiaoli Wen, Jing Wan, Qizhi He, Mengfei Wang, Shuangdi Li, Mei Jiang, Zhen Qian, Binya Liu, Wen Lu, Kai Wang, Kun Gao, Xiaoping Wan

**Affiliations:** 1 Department of Gynecology, Shanghai First Maternity and Infant Hospital, Tongji University School of Medicine, Shanghai, 201204 China; 2 Department of Gynecology, International Peace Maternity and Child Health Hospital, Shanghai Jiao Tong University School of Medicine, Shanghai, 200030 China; 3 Department of Pathology, Shanghai First Maternity and Infant Hospital, Tongji University School of Medicine, Shanghai, 201204 China; 4 Tongji University School of Medicine, Shanghai, 200092 China; 5 Clinical and Translational Research Center, Shanghai First Maternity and Infant Hospital, Tongji University School of Medicine, Shanghai, 201204 China

**Keywords:** Gynaecological cancer, Cancer genetics

## Abstract

The Rho family of GTPases is strictly regulated by a large family of GTPase-activating proteins (GAPs) that stimulate the relatively weak intrinsic GTP-hydrolyzing activity of Rho GTPases. p190A is a potent and widely expressed GAP that acts on RhoA GTPases. p190A is frequently mutated in endometrial cancer, but the contribution of p190A mutations to endometrial tumorigenesis remains unclear. Here we identified that p190A is an upstream regulator of the Hippo-YAP signaling pathway, which is a critical regulator of cell proliferation, apoptosis, and cell fate. p190A knockout in endometrial cancer cells promoted cell proliferation, migration, and epithelial–mesenchymal transition (EMT), which were partially dependent on YAP activation. Wild-type p190A, but not endometrial cancer-associated mutants, suppressed the nuclear localization, transcriptional activity, and malignant transformation function of YAP. Moreover, the nuclear localization of YAP was enhanced in p190A-mutated endometrial cancer. These findings reveal novel molecular mechanisms underlying Hippo-YAP pathway-driven endometrial tumorigenesis and elucidate the potential for therapy targeting the Hippo-YAP pathway in p190A-mutated endometrial cancer.

## Introduction

Endometrial cancer is the sixth most common neoplasm in females worldwide and causes ∼74,000 deaths per year.^1^ Previous large-scale sequencing studies on primary endometrial cancer samples indicated that samples with phenotype differences can be classified into distinct molecular subgroups with further molecular subclustering.^2^ Four distinct molecular subgroups, POLE-mutated (ultramutated), microsatellite unstable (hypermutated), copy number low (endometrioid), and copy number high (serous-like), were distinguished in endometrial cancer and analyzed using The Cancer Genome Atlas (TCGA) dataset.^2^ Endometrioid carcinomas populate all four molecular subgroups, whereas serous carcinomas are almost exclusively found in the copy number high (serous-like) subgroup.^2^ This molecular classification provides a method for observing and describing endometrial cancers, while providing important information on diagnostic, prognostic, and therapeutic targets.^2^

Rho GTPases regulate cytoskeletal and cell adhesion dynamics and thereby coordinate the cellular responses required for cell migration, cell polarity, and cell cycle progression.^3,4^ RhoA is one of the most well-characterized Rho GTPase family members and has long been involved in malignant transformation, tumor invasion, and metastasis.^5^ The activity of Rho GTPases is regulated by GTPase-activating proteins (GAPs) and guanine nucleotide-exchange factors (GEFs). The former enhances the relatively slow intrinsic GTPase activity of Rho proteins, whereas the latter catalyzes the exchange of GDP for GTP in vivo.^5^ Rho activity is suppressed by GAPs and promoted by GEFs. p190A RhoGAP (hereafter p190A) is the most widely studied RhoGAP and is generally regarded as the main RhoGAP for RhoA in cells.^6^ p190A is also associated with cell migration, epithelial differentiation, polarity, cell–cell junctions, and cell division.^6^ Importantly, *ARHGAP35*, the gene encoding p190A, was found to be frequently mutated in up to 15% of endometrial cancers and 2% of all tumors in a studied cohort.^7,8^

The Hippo pathway is an evolutionarily conserved kinase signaling cascade that includes MST and LATS kinases and results in YAP/TAZ phosphorylation, cytoplasmic retention, and inhibition.^9^ Physiological or pathological inactivation of these kinases leads to YAP/TAZ dephosphorylation, nuclear accumulation, and target gene transactivation. Activated nuclear YAP/TAZ binds to the TEA/ATTS domain transcription factors (TEAD1–4) to mediate the expression of target genes.^9^ The Hippo pathway is a key regulator of various biological processes, such as tissue homeostasis, cell proliferation, and apoptosis.^9^ Considering the critical roles of the YAP/TAZ-TEAD complex in cancer initiation, progression, metastasis, and recurrence, these proteins and their upstream regulators could serve as potential therapeutic targets.^10^

Although p190A mutations in endometrial cancers have been described in several cancer sequencing studies,^2,7,8,11^ the clinical relevance and biological functional impact of p190A mutations remain poorly understood. To address these issues, we analyzed the latest endometrial cancer TCGA cohort and demonstrated that p190A was frequently mutated and downregulated in endometrial cancers. p190A mutations frequently co-occurred with POLE mutations. Mechanistically, we demonstrated that p190A inactivation in endometrial cancer cells led to malignant transformation by aberrant activation of the Hippo-YAP pathway outputs. Our data revealed that p190A acts as a tumor suppressor in endometrial cancer and suggested that inhibition of YAP activity with small-molecule inhibitors represents a potential therapeutic opportunity for p190A-mutated endometrial cancer.

## Results

### p190A is frequently mutated and downregulated in endometrial cancer

**Fig. 1:**
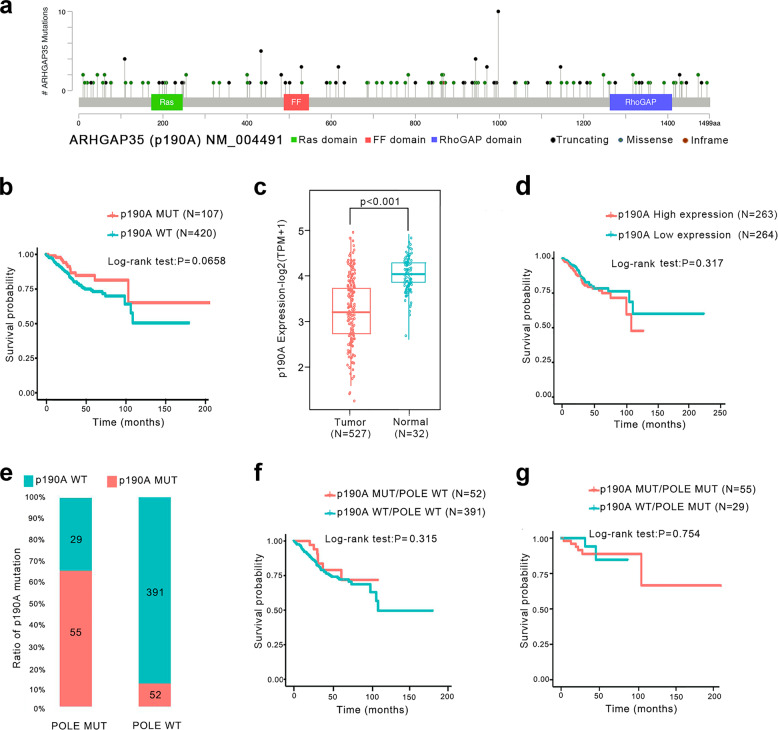
p190A is frequently mutated and downregulated in the TCGA cohort of endometrial cancer patients. **a** Schematics of the p190A proteins show the positions of individual somatic mutations identified in the endometrial cancer TCGA cohort. **b** Kaplan–Meier survival curves for p190A-mutated and p190A wild-type endometrial cancer patients from the TCGA cohort. **c** The relative mRNA expression of p190A in normal endometrial tissues and endometrial cancer tissues from the TCGA cohort. **d** Kaplan–Meier survival curves for patients with high vs. low mRNA expression of p190A in the endometrial cancer TCGA cohort. High- and low-expression groups were defined by the median p190A mRNA expression value of the study population. Patients whose tumors had p190A expression above the median value were termed “high expression”, whereas those whose tumors had p190A expression below the median were termed “low expression.” **e** The co-occurrence of p190A and POLE mutations in the endometrial cancer TCGA cohort. Only pathogenic POLE mutations that occurred in the exonuclease domain were included. **f** Kaplan–Meier survival curves for p190A-mutated/POLE wild-type and p190A/POLE-double wild-type endometrial cancer patients in the TCGA cohort. **g** Kaplan–Meier survival curves for p190A wild-type/POLE-mutated and p190A/POLE-double mutated endometrial cancer patients in the TCGA cohort

Recurrent mutation of p190A was found in endometrial cancer;^2,7,8,11^ however, its clinical implication remains poorly understood. To address this issue, we performed bioinformatics analyses of the endometrial cancer TCGA dataset downloaded from the CBioPortal database. We finally obtained a total of 527 cases matched with whole-exome sequencing (WXS) data and transcriptome profiles (RNA sequencing, RNA-Seq), as well as clinical information. A total of 152 p190A mutations occurred in approximately 20.3% (107/527) of the tumor specimens, including 76 truncating mutations, 74 missense mutations, and 2 inframe mutations (Fig. 1a). These mutations were nearly evenly distributed throughout the coding sequence (Fig. 1a). The high incidence of truncating mutations implied that p190A might be inactivated in endometrial cancer. In terms of the potential prognostic value of the p190A mutations in endometrial cancer, patients with p190A mutations tended to have a better prognosis than those with the wild-type p190A gene, although the trend was not statistically significant (*p* = 0.0658) (Fig. 1b). Moreover, p190A mutation status was not associated with clinicopathologic variables, such as tumor histological type, histological grade, or clinical stage (data not shown). We then analyzed the RNA-Seq data of endometrial cancer specimens and normal endometrial tissues from the TCGA cohort. The levels of p190A transcripts were significantly lower in endometrial cancer specimens than in normal tissues (*p* < 0.001) (Fig. 1c). However, the mRNA expression of p190A was not associated with the overall survival of cancer patients (*p* = 0.317) (Fig. 1d). A previous study revealed that the survival benefit of ultramutated tumors with POLE mutations was profound compared with other subtypes of endometrial cancer.^2^ Interestingly, the co-occurrence of p190A and POLE mutations was observed in endometrial cancer (Fig. 1e). However, P190A mutations were not a favorable factor in the POLE wild-type (*p* = 0.315) (Fig. 1f) or POLE-mutated (*p* = 0.754) (Fig. 1g) subgroup. Taken together, these results suggest that p190A is frequently mutated and downregulated in endometrial cancer. More patient data from multicenter studies are warranted to further validate and assess whether the p190A mutation represents a favorable prognostic factor in endometrial cancer.

### p190A suppresses endometrial cancer cell growth and migration

**Fig. 2:**
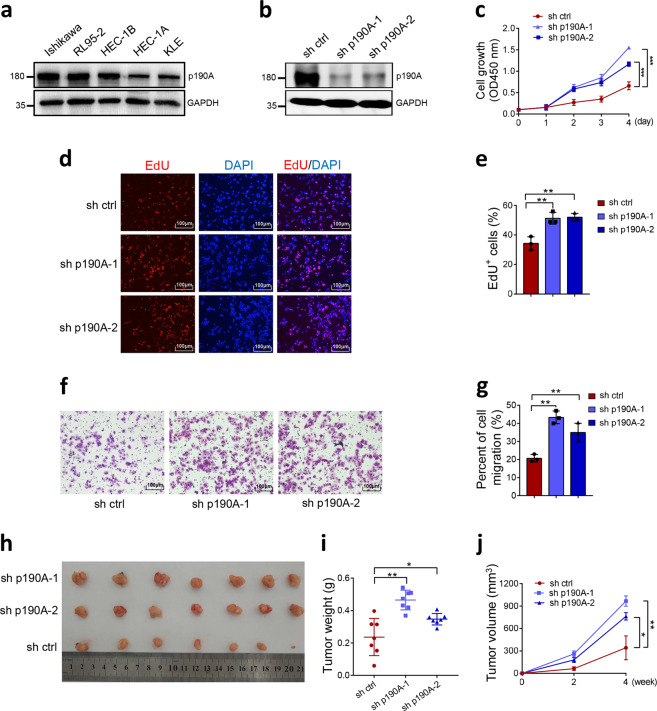
p190A knockdown promotes cell proliferation and migration in endometrial cancer cells. **a** Western blotting of the indicated proteins in WCLs (whole cell lysates) from five endometrial cancer lines. **b** Western blotting of the indicated proteins in WCLs from Ishikawa cells stably expressing sh ctrl, sh p190A-1, or sh p190A-2. **c** CCK-8 cell proliferation analysis of Ishikawa cells stably expressing sh ctrl, sh p190A-1, or sh p190A-2. Data are shown as the mean ± SD (*n* = 3). **d**, **e** EdU incorporation analysis of Ishikawa cells stably expressing sh ctrl, sh p190A-1, or sh p190A-2 (**d**), and the quantitative data are shown in **e**. Data are shown as the mean ± SD (*n* = 3). Scale bar, 100 μm. **f**, **g** Transwell migration analysis of Ishikawa cells stably expressing sh ctrl, sh p190A-1, or sh p190A-2 (**f**), and the quantitative data are shown in **g**. Data are shown as the mean ± SD (*n* = 3). Scale bar, 100 μm. **h** Image of Ishikawa cells stably expressing sh ctrl, sh p190A-1, or sh p190A-2-implanted tumors from nude mice. **i** The weight of xenograft tumors shown in **h**. *n* = 7. **j** The tumor volume of xenograft tumors measured on the indicated days after injection

We examined p190A protein expression in several endometrial cancer cell lines and found that p190A was ubiquitously expressed (Fig. 2a). According to the WXS data provided by Cancer Cell Line Encyclopedia (https://portals.broadinstitute.org/ccle), p190A is wild-type in Ishikawa, KLE, and RL95-2 cells but mutated in HEC-1A and HEC-1B cells. We verified that p190A in Ishikawa, KLE, and RL95-2 cells is wild-type by Sanger sequencing of the exons (data not shown). We depleted p190A expression in Ishikawa cells using two independent short hairpin RNAs (shRNAs) and then examined the key features of cancer cell lines (Fig. 2b). Cell Counting Kit-8 (CCK-8) and colony-formation assays showed that p190A depletion markedly increased Ishikawa cell growth (Fig. 2c and Supplementary Fig. 1a, b). The 5-ethynyl-2’-deoxyuridine (EdU) incorporation assay showed that p190A-depleted Ishikawa cells displayed a substantial increase in DNA synthesis compared with that in control cells (Fig. 2d, e). In addition, Transwell and wound-healing assays revealed that p190A depletion markedly increased Ishikawa cell migration (Fig. 2f, g and Supplementary Fig. 1c, d). Furthermore, compared with the control, p190A depletion markedly accelerated Ishikawa xenograft tumor growth (Fig. 2h–j). The tumor-suppressive activity of p190A was also observed in another two endometrial cancer cell lines, KLE and RL95-2 (Supplementary Figs. 2 and 3). Taken together, these results suggest that p190A suppresses endometrial cancer cell growth and migration in vitro and tumor growth in vivo.

### p190A knockout in endometrial cells leads to EMT

**Fig. 3:**
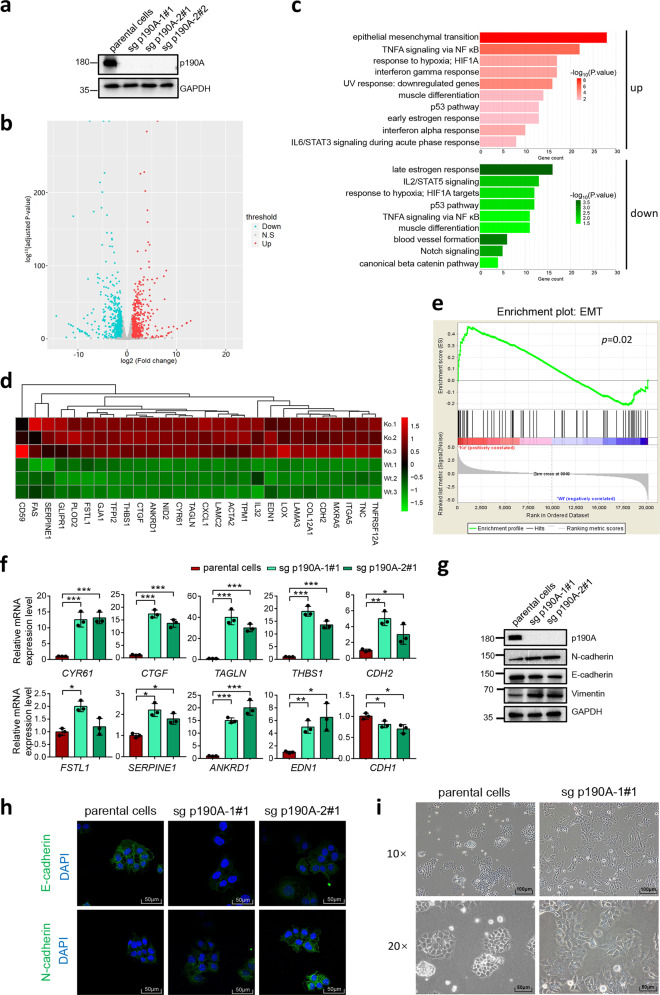
p190A KO in Ishikawa cells induces molecular and morphologic changes indicative of EMT. **a** Western blotting of the indicated proteins in WCLs from Ishikawa cells with p190A KO by CRISPR-Cas9 methods. Parental Ishikawa cells were used as a control. **b** Volcano plot of the differentially expressed genes in parental and p190A-KO Ishikawa cells. **c** KEGG pathway analysis of the differentially expressed genes in parental and p190A-KO Ishikawa cells. **d** Heatmap depicting the expression of 28 differentially expressed EMT-related genes in parental and p190A-KO Ishikawa cells. **e** GSEA of the EMT gene signature in parental and p190A-KO Ishikawa cells. The hallmark EMT gene set (Standard name: JECHLINGER_EPITHELIAL_TO_MESENCHYMAL_TRANSITION _UP) was obtained from the Molecular Signatures Database (MsigDB). **f** RT-qPCR measurement of the mRNA expression of EMT-related genes in parental and p190A-KO Ishikawa cells. Data are shown as the mean ± SD (*n* = 3). **g** Western blotting of the indicated proteins in WCLs from parental and p190A-KO Ishikawa cells. **h** E-cadherin and N-cadherin were analyzed by immunofluorescence in parental and p190A-KO Ishikawa cells. Scale bar, 50 μm. **i** The morphological changes are shown in the phase contrast images. Phase contrast ×10 and ×20 images of parental and p190A-KO Ishikawa cells. Scale bar, 100 μm

To gain further insight into the molecular link between p190A inactivation and the pathogenesis of endometrial cancer, we investigated the global transcriptomic change in p190A-depleted Ishikawa cells by RNA-Seq. To completely ablate p190A protein expression, we generated p190A knockout (KO) cell lines using CRISPR/Cas9 methods with two different sgRNAs. As shown in Fig. 3a and Supplementary Fig. 4, p190A protein expression was completely ablated in several Ishikawa cell clones. RNA-Seq was then performed to determine the differentially expressed genes between parental Ishikawa cells and the pools mixed with three independent p190A-KO clones. A total of 931 protein-coding genes showed altered expression in p190A-KO Ishikawa cells compared with control cells (Fig. 3b). A total of 440 genes were upregulated (>2-fold), whereas 491 genes were downregulated (<2-fold) (Supplementary Table 1). To investigate the functional association of the differentially expressed genes, we conducted Kyoto Encyclopedia of Genes and Genomes (KEGG) pathway enrichment analysis to identify the most significant pathways that were altered in p190A-KO Ishikawa cells. The results showed that multiple signaling pathways (e.g., EMT, tumor necrosis factor-α signaling, hypoxia response, estrogen response, and interferon (IFN) α/γ response) were significantly altered, suggesting that p190A was likely to interfere with these pathways (Fig. 3c). In this study, we focused on the potential role of p190A in EMT, an evolutionarily conserved developmental process that has been implicated in tumorigenesis and confers metastatic features upon cancer cells by enhancing migration, invasion, and apoptotic resistance.^12^ Gene set enrichment analysis (GSEA) showed that p190A KO upregulated the EMT gene signature in Ishikawa cells (Fig. 3d, e). The mRNA levels of EMT-related genes (e.g., *CDH2* (N-cadherin), *CYR61*, *CTGF*, *THBS1*, *FSTL1*, and *ANKRD1*) were markedly upregulated by p190A ablation (Fig. 3f). The mRNA level of *CDH1* (E-cadherin), a well-known EMT repressor, was moderately downregulated in p190A-KO Ishikawa cells (Fig. 3f). Western blotting and immunofluorescence (IF) showed that N-cadherin expression was upregulated, whereas E-cadherin was downregulated in p190A-KO Ishikawa cells (Fig. 3g, h). Moreover, p190A ablation induced a dramatic morphological change in p190A-KO cells. Although parental Ishikawa cells exhibited the typical cobblestone epithelial morphology, the p190A-KO Ishikawa cells presented an elongated and fibroblastic morphology with reduced cell–cell contacts (Fig. 3i). p190A depletion also led to the elevation of EMT markers in KLE and RL95-2 cells (Supplementary Fig. 5). Collectively, these data demonstrate that p190A inactivation in endometrial cancer cells induces molecular and morphologic changes that are indicative of EMT.

### p190A KO leads to aberrant activity of the Hippo-YAP pathway

**Fig. 4:**
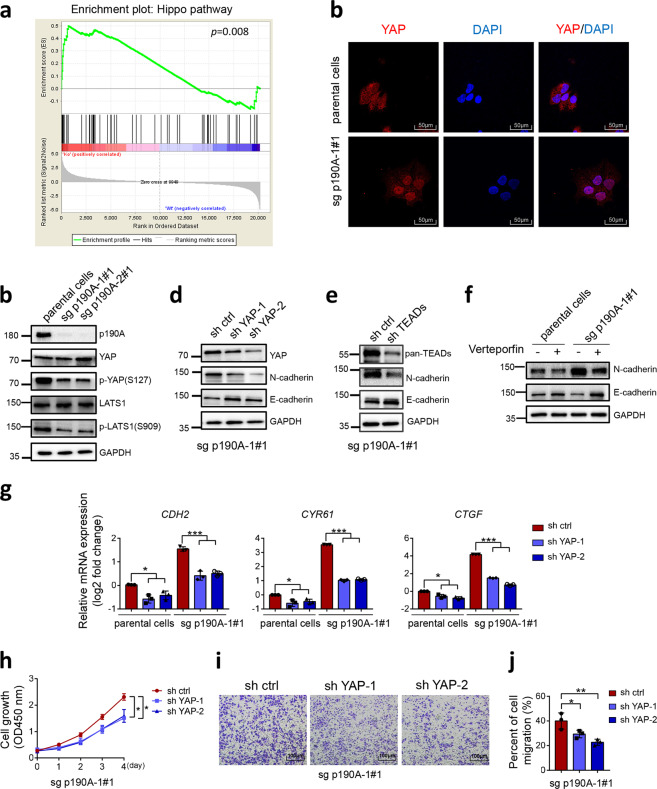
KO of p190A in Ishikawa cells leads to aberrant Hippo-YAP pathway activity. **a** GSEA of the Hippo pathway gene signature in parental and p190A-KO Ishikawa cells. The hallmark Hippo pathway gene set (Standard name: CORDENONSI_YAP_CONSERVED_ SIGNATURE) was obtained from MsigDB. **b** Western blotting of the indicated proteins in WCLs from parental and p190A-KO Ishikawa cells. **c** YAP localization was analyzed by immunofluorescence in parental and p190A-KO Ishikawa cells. Scale bar, 50 μm. **d** Western blotting of the indicated proteins in WCLs from p190A-KO Ishikawa cells stably expressing sh ctrl, sh YAP-1, or sh YAP-2. **e** Western blotting of the indicated proteins in WCLs from p190A-KO Ishikawa cells stably expressing sh ctrl or sh TEADs. **f** Western blotting of the indicated proteins in WCLs from p190A-KO Ishikawa cells treated with verteporfin (2 μM) for 48 h. **g** RT-qPCR measurement of the mRNA expression of EMT-related genes in parental and p190A-KO Ishikawa cells stably expressing sh ctrl, sh YAP-1, sh YAP-2. Data are shown as the mean ± SD (*n* = 3). **h** CCK-8 cell proliferation analysis of parental and p190A-KO Ishikawa cells stably expressing sh ctrl, sh YAP-1, or sh YAP-2. Data are shown as the mean ± SD (*n* = 3). **i**, **j** Transwell migration analysis of parental and p190A-KO Ishikawa cells stably expressing sh ctrl, sh YAP-1, or sh YAP-2 (**i**), and the quantitative data are shown in **j**. Data are shown as the mean ± SD (*n* = 3). Scale bar, 100 μm

The Hippo-YAP pathway plays a critical role in EMT.^13^ We noticed that several p190A-regulated EMT-related genes, such as *CTGF*, *CYR61*, and *ANKRD1*, are well-characterized YAP transcriptional targets. GSEA showed that p190A KO upregulated the Hippo transcriptional target gene signature in Ishikawa cells (Fig. 4a). To evaluate whether the EMT induced by p190A KO is dependent on aberrant YAP activation, we first investigated the impact of p190A KO on YAP phosphorylation and localization. Compared with the control, p190A ablation decreased the phosphorylation levels of LATS1 and YAP in Ishikawa cells (Fig. 4b). In sparsely growing parental Ishikawa cells, YAP was diffusely localized throughout the cell, as detected by IF analysis (Fig. 4c). By contrast, intense nuclear YAP staining was observed in p190A-KO Ishikawa cells. p190A depletion also led to a decrease in LATS1/YAP phosphorylation in KLE and RL95-2 cells (Supplementary Fig. 5a, c). Thus, these results suggest that p190A negatively regulates YAP activity.

To further validate that EMT induced by p190A inactivation occurs via the Hippo-YAP pathway, we stably depleted YAP expression in p190A-KO Ishikawa cells by two independent shRNAs. YAP depletion upregulated E-cadherin but downregulated N-cadherin in p190A-KO Ishikawa cells compared with control cells (Fig. 4d). A similar result was observed in p190A-KO Ishikawa cells depleted of TEAD proteins by shRNA targeting TEAD1/3/4 (Fig. 4e). Changes in E/N-cadherin protein levels by p190A KO were partially reversed by treatment with verteporfin,^14^ a small-molecule inhibitor of YAP-TEAD binding (Fig. 4f). Moreover, depletion of YAP or TEADs reduced the mRNA expression of EMT-related genes (Fig. 4g and Supplementary Fig. 6a), cell proliferation (Fig. 4h and Supplementary Fig. 6b), and migration (Fig. 4i, j and Supplementary Fig. 6c, d) in p190A-KO Ishikawa cells. Verteporfin treatment also reversed the pro-oncogenic phenotypes observed in p190A-KO Ishikawa cells (Supplementary Fig. 6e–h). Taken together, these results indicate that p190A KO leads to EMT, at least in part, through activation of Hippo-YAP transcriptional outputs in endometrial cancer cells.

### Endometrial cancer-associated mutations impair the tumor-suppressive functions of p190A

**Fig. 5:**
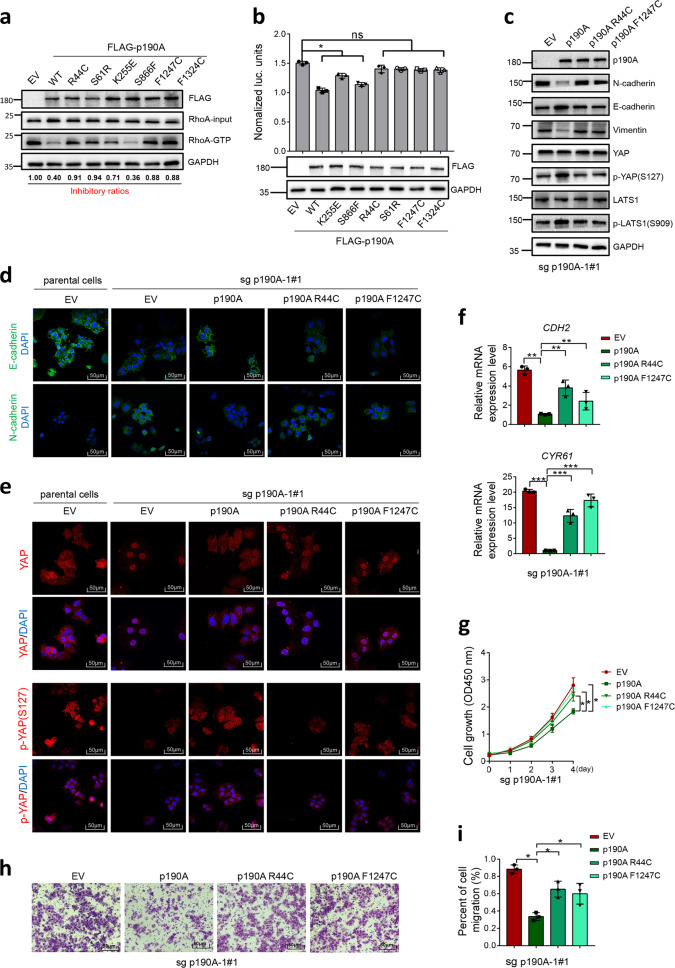
Endometrial cancer-associated p190A mutants lose their tumor-suppressive function. **a** Active RhoA protein levels were measured in 293T cells expressing EV, p190A-WT or a panel of endometrial cancer-associated mutants of p190A by a GST-RBD pull-down assay. The relative signal ratios [RhoA-GTP × FLAG-p190A mutant/ (input RhoA × FLAG-p190A-WT)] were calculated and normalized to EV [RhoA-GTP (EV)/(input RhoA (EV)]. **b** Serum response factor (SRF) transcriptional activities were measured in 293T cells expressing EV, p190A-WT, or a panel of endometrial cancer-associated mutants of p190A by an SRE-Luc reporter assay. The corresponding WB is shown in the lower panel. **c** Western blotting of the indicated proteins in WCLs from p190A-KO Ishikawa cells stably expressing EV, p190A-WT, or endometrial cancer-associated mutants (R44C, F1247C). **d** Immunofluorescence analysis of E/N-cadherin in parental or p190A-KO Ishikawa cells stably expressing EV, p190A-WT, or endometrial cancer-associated mutants (R44C, F1247C). Scale bar, 50 μm. **e** Immunofluorescence analysis of total or phospho-YAP in parental or p190A-KO Ishikawa cells stably expressing EV, p190A-WT or endometrial cancer-associated mutants (R44C, F1247C). Scale bar, 50 μm. **f** RT-qPCR measurement of the mRNA expression of EMT-related genes in parental or p190A-KO Ishikawa cells stably expressing EV, p190A-WT, or endometrial cancer-associated mutants (R44C, F1247C). Data are shown as the mean ± SD (*n* = 3). **g** CCK-8 cell proliferation analysis of p190A-KO Ishikawa cells stably expressing EV, p190A-WT, or endometrial cancer-associated mutants (R44C, F1247C). Data are shown as the mean ± SD (*n* = 3). **h**, **i** Transwell migration analysis of p190A-KO Ishikawa cells stably expressing EV, p190A-WT, or endometrial cancer-associated mutants (R44C, F1247C), and the quantitative data are shown in **i**. Data are shown as the mean ± SD (*n* = 3). Scale bar, 50 μm

The *p190A* gene is one of the most recurrently mutated genes in endometrial cancer.^2,7,8^ However, the downstream pathways affected by p190A mutants and their roles in the oncogenic phenotypes of endometrial cancer remain limited. Given that p190A is a major RhoGAP toward RhoA in mammalian cells, we hypothesized that p190A loss-of-function mutations may lead to aberrant activation of RhoA and its downstream signaling. We first confirmed that p190A was an inhibitor of RhoA-GTP in endometrial cancer cells: p190A depletion increased the active RhoA level, as assessed by the Rho binding domain (RBD) pull-down assay (Supplementary Fig. 7a), and the intensity of phospho-MLC (surrogate marker for RhoA activity), as indicated by IF analysis (Supplementary Fig. 7b). Approximately half of p190A mutations are truncating mutations that may produce no functional protein products. Alternatively, the mutated p190A mRNAs may be degraded via the nonsense-mediated mRNA decay pathway.^15^ Thus, we focused on whether the missense mutations of p190A could impair their RhoGAP activities and tumor-suppressive functions. The RBD pull-down results showed that overexpression of wild-type p190A in 293T cells decreased the amount of active RhoA compared with that in control cells and all endometrial cancer-associated p190A mutants, except p190A-S866F, showed impaired RhoGAP activities (Fig. 5a). Similar results were obtained by using another Rho activation detection assay (SRE-Luc reporter) to assess the effect of wild-type or p190A mutants on RhoA downstream serum response factor activities (Fig. 5b). We next examined the functional impact of p190A mutants on EMT and YAP activity. p190A-KO Ishikawa cells were reconstituted with p190A-WT or endometrial cancer-associated p190A mutants (R44C or F1247C). Ectopic-expressed p190A-WT, but not endometrial cancer-associated p190A mutants, elevated E-cadherin and reduced N-cadherin expression, as demonstrated by western blotting and IF analysis (Fig. 5c, d). Similarly, ectopically expressed wild-type p190A, but not endometrial cancer-associated p190A mutants, elevated LATS1/YAP phosphorylation and reduced nuclear YAP localization (Fig. 5c, e). Moreover, the increase in EMT gene expression, cell growth, and migration caused by p190A KO were reversed by ectopic expression of wild-type p190A, whereas endometrial cancer-associated p190A mutants had no effects (Fig. 5f–i). These results suggest that endometrial cancer-associated p190A mutations may impair their RhoGAP activities, favoring the oncogenic transformation of endometrial cancer cells.

### YAP is activated in samples of p190A-mutated endometrial cancer tissues

**Fig. 6:**
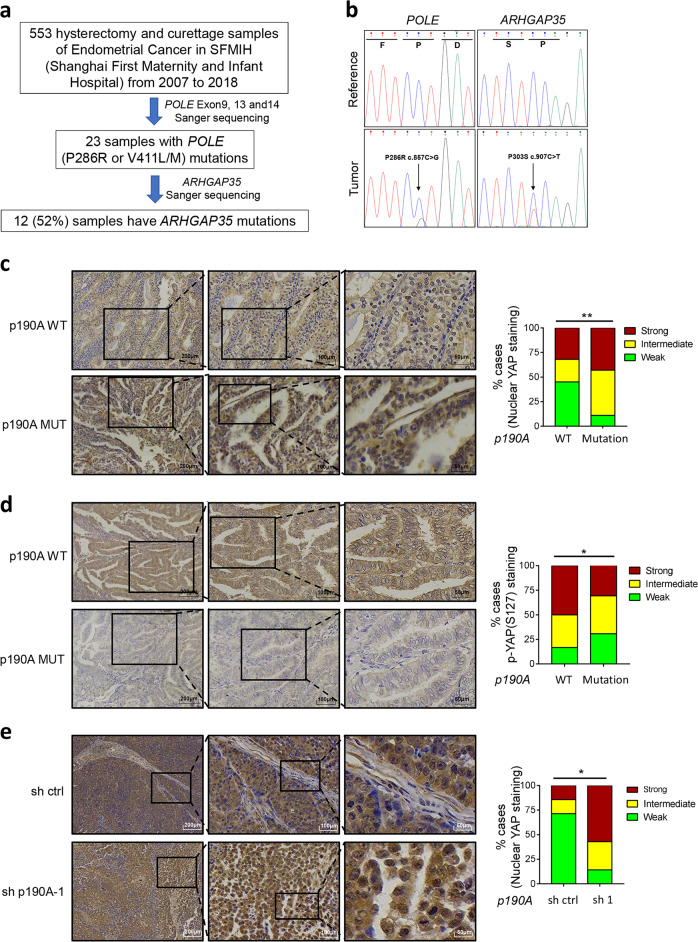
p190A inactivation increases YAP nuclear accumulation in both endometrial cancer specimens and Ishikawa xenograft tumors. **a** Flow chart of the mutation screens performed to identify POLE and ARHGAP35 (p190A) mutations in endometrial cancer specimens. **b** Sanger sequencing confirming that POLE or ARHGAP35 was mutated in endometrial cancer specimens. **c**, **d** Left, immunohistochemical analysis of YAP (**c**) and phospho-YAP (S127) (**d**) expression in endometrial cancer specimens. The inset in each panel shows a high magnification image of the representative (framed) area. Right, the quantitative data indicate the intensity of nuclear YAP (**c**) and phospho-YAP (S127) (**d**) staining. Scale bar, 100 μm. **e** Left panel, immunohistochemical analysis of YAP expression in Ishikawa xenograft tumors. The inset in each panel shows a high magnification image of the representative (framed) area. Right, the quantitative data indicate the intensity of nuclear YAP staining. Scale bar, 100 μm

To further determine whether YAP is activated in p190A-mutated endometrial cancer, we examined YAP localization and phosphorylation in formalin-fixed, paraffin-embedded (FFPE) endometrial cancer specimens. Considering the genetic heterogeneity of endometrial cancer and the co-occurrence of p190A and POLE mutations in endometrial cancer, we analyzed the YAP status in p190A-WT/POLE-MUT or p190A-MUT/POLE-MUT endometrial cancer specimens. We collected 553 primary endometrial cancer specimens and performed Sanger sequencing for exons 9, 13, and 14 of POLE (exonuclease proofreading domain). Twenty-three samples with POLE hotspot mutations at P286R or V411L/M were detected. We then performed Sanger sequencing for all protein-coding exons of p190A (Fig. 6a, b). Truncating or missense mutations of p190A were detected in 12 samples and the remaining 11 samples were p190A wild type (Supplementary Table 2). Immunohistochemistry (IHC) analysis was performed to detect the level of total YAP or phospho-YAP (S127) in 23 cancer specimens (Fig. 6c, d). The nuclear YAP intensity was assessed as weak, moderate, or strong. When YAP localization data were compared with the mutation status of p190A, a strong correlation was observed between p190A mutations and YAP nuclear localization (Fig. 6c). Similarly, a strong correlation was found between p190A mutations and phospho-YAP (S127) intensity (Fig. 6d). Last, YAP nuclear localization was enhanced in p190A-KD Ishikawa xenografts compared with sh control xenografts (Fig. 6e). These results indicate that p190A mutations are associated with YAP activation in endometrial cancer specimens, supporting a possible pathological role of the Hippo-YAP pathway in p190A mutation-induced carcinogenesis.

## Discussion

Previous large-scale sequencing studies of endometrial cancer detected an unexpectedly high frequency of loss-of-function mutations in p190A.^7,8^ RhoA GTPase was previously reported as a positive upstream regulator of YAP activity via its inhibition of LATS1/2 kinases.^16^ In our study, we revealed that YAP activation represents an important molecular event contributing to p190A inactivation-induced endometrial carcinogenesis, which is dependent on the aberrant activation of RhoA GTPases (Supplementary Fig. 8). Furthermore, we demonstrated that YAP activation may be involved, at least in part, in p190A KO-induced cell growth, migration, and EMT. In a recent study, Frank et al.^17^ showed that p190A induced contact inhibition of cell proliferation in Madin-Darby Canine Kidney cells by repressing the transcription of YAP target genes through the RhoA-LATS-YAP cascade, suggesting that the impact of p190A on the Hippo-YAP pathway is not limited to endometrial cancer-derived cells.

In this study, the co-occurrence of p190A and POLE mutations was observed in endometrial cancer. Approximately 7–12% of endometrial cancer with somatic mutation in the proofreading exonuclease domain of the POLE gene.^2^ This POLE mutation is associated with the disruption of the exonuclease activity required for proofreading function and results in a high mutational burden or “ultramutator” phenotype.^18^ Thus, the particularly high mutation rates of p190A in endometrial cancer may be caused, at least in part, by POLE proofreading defects. Our results showed that patients bearing p190A mutations showed a potential trend of better prognosis than those with the wild-type p190 gene. Recent studies reported that POLE-mutated endometrial cancer was characterized by robust tumor T-cell infiltration. This phenomenon provides a plausible explanation for the better survival in these cancer patients.^19,20^ Considering that the immune-related interleukin-6/signal transducer and activator of transcription 3 and IFNα/β pathways were markedly elevated in p190A-KO endometrial cancer cells, the cytotoxic T-lymphocyte infiltration status should be investigated in p190A-mutated endometrial cancer.

RhoA is a well-studied Rho GTPase and has long been implicated in malignant transformation, tumor invasion and metastasis.^3,21^ Activating RhoA mutations are found in a subset of diffuse-type gastric cancer and lymphoma but very rare in most human cancers.^22^ Therefore, inactivating mutations in the negative regulators of RhoA may represent alternative ways to achieve constitutive activation of RhoA. The human genome encodes more than 70 members of the RhoGAP family. Several RhoGAPs, such as DLC1 and DLC2, are downregulated in various human cancers,^23^ but only p190A has been identified as a significantly mutated gene in human cancers. Thus, p190A may play critical roles in tumorigenesis. We found that the mRNA level of p190A was downregulated in endometrial cancer by analyzing the RNA-seq data from TCGA. Whether epigenetic alterations, such as DNA methylation or histone modifications, are involved in the downregulation of p190A mRNA expression in a subset of p190A wild-type endometrial cancers should be investigated in future studies.

Approximately half of endometrial cancer-associated p190A mutations are truncating mutations indicative of loss of function. In contrast, the functional impacts of missense and inframe p190A mutations are still poorly understood. Our results showed that the majority of endometrial cancer-associated p190A mutants, except p190A-S866F, showed impaired RhoGAP activities. Similarly, Frank et al.^17^ showed that the p190A-R1284K mutant was unable to restore contact inhibition in cells depleted of p190A and p190B. However, another study found that endometrial cancer-associated p190A mutants that occurred in the PLS domain even had higher RhoGAP activities than wild-type p190A. Binamé et al.^24^ showed that gain-of-function mutations, such as S866F and Δ865–870, favored random cell migration but decreased directed migration, which may lead to an enhanced exploratory behavior of tumor cells. Therefore, different p190A mutations may have divergent effects on p190A activities, and not all p190A mutations can lead to activation of the RhoA-YAP axis in endometrial cancer.

Our bioinformatic and functional data established a new tumor-suppressive pathway in endometrial cancer. Our findings also suggested that targeting YAP activity with small molecular inhibitors may represent a potential therapeutic strategy for p190A-mutated endometrial cancer. Much remains to be done to evaluate whether p190A mutations promote malignant transformation of endometrial cancer cells through pathways other than the RhoA-YAP axis, given that RhoA-independent functions of p190A have been reported.^25,26^ It will also be useful to generate mouse models of conditional endometrial-specific p190A KO to further investigate p190A-null phenotypes in vivo and determine whether the Hippo-YAP pathway is indispensable for p190A mutation-induced endometrial cancer.

## Materials and methods

### TCGA data acquisition and reprocessing

The expression profiles, somatic mutation data, and clinical information of endometrial cancers were obtained via cBioPortal (https://www.cbioportal.org/). The expression profiles of normal endometrial tissues were obtained via GDC Data Portal (https://portal.gdc.cancer.gov/projects/TCGA-UCEC). The normalization and differential expression analysis of the transcriptome data were conducted by the edgeR package. Moreover, the R package survminer was used to compare the survival differences between the two groups based on the log-rank test.

### Cell culture and transfection

293T, RL95-2, Ishikawa, HEC-1A, HEC-1B, and KLE cells were maintained in Dulbecco’s modified Eagle medium (DMEM) with 10% (v/v) fetal bovine serum (FBS). Sanger sequencing results verified that RL95-2, Ishikawa, and KLE cells are p190A wild-type cell lines. Lipofectamine 2000 (Thermo, USA) was used for transient transfection.

### Expression constructs

The p190A coding sequence (CDS) was amplified from the HeLa cDNA library and subcloned into the pCMV-FLAG vector (Clontech, USA). Endometrial cancer-associated p190A mutants were generated by using the KOD-Plus-Mutagenesis Kit (TOYOBO, Japan) following the manufacturer’s instructions.

### Lentivirus infection and stable cell generation

The shRNAs targeting p190A werewas subcloned into the pLKO.3G plasmid (Addgene, USA). The viruses were collected from the medium at 48 h after transfection. For knockdown experiments, cells were infected with viruses over 48 h in the presence of polybrene. FLAG-p190A or its mutants were subcloned into the PCDH plasmid (System Bioscience, USA) for protein overexpression. The shRNA sequences are listed in Supplementary Table 3.

### Cell proliferation assays

Cell proliferation was determined using the CCK-8 Kit (Dojindo, Japan) following the manufacturer’s instructions. Cells were seeded in 96-well plates at a density of 1 × 10^3^ cells/well. After 1- to 4-day culture periods, 10 μl CCK-8 solution was added to the cell culture and the cells were incubated for 2 h. The resulting color was measured on a Multimode Plate Reader (Molecular Devices, USA) at 450 nm. Each treatment was performed in triplicate and experiments were repeated over three times.

### EdU assays

A EdU incorporation assay was performed to investigate DNA synthesis using an EdU Cell Proliferation Kit with Alexa Fluor 555 (Beyotime, China). Cells were seeded in six-well plates. The EdU incorporation experiments were performed following the manufacturer’s instructions. Nuclei were stained with 4′,6-diamidino-2-phenylindole (DAPI). The cells were visualized using a confocal microscope (Olympus, Japan).

### Migration assays

Cell migration was determined by using the Transwell (Corning, USA) migration assay. Cells were precultured in serum-free medium for 48 h. A total of 3 × 10^4^ cells were seeded in serum-free medium on the upper chamber and the lower chamber was filled with DMEM containing 5% FBS. After 48 h, the nonmigrated cells on the upper chambers were carefully removed with a cotton swab and the cells adhered to the bottom were stained with 0.5% crystal violet. The positive cells were visualized using a microscope.

### Mouse xenograft assays

The animal study was approved by the Ethics Review Committee for Animal Experimentation of Tongji University. BALB/c nu/nu mice (4–6 weeks old) were housed in our institutional pathogen-free mouse facilities. Ishikawa cells (5 × 10^6^) were suspended in 100 μl of PBS buffer and injected into the flanks of the female nude mice (seven mice in each group). At the end of 4 weeks, the mice were killed and the tumor xenografts were collected and weighed.

### CRISPR/Cas9-mediated gene knockout

The guide oligos targeting the p190A gene were subcloned into the pX459 plasmid (Addgene, USA). After 24 h, cells were cultured in 1 µg/ml puromycin for 3 days. Surviving cells were seeded in a 96-well plate by limited dilution to isolate the monoclonal cell lines. KO cell clones were detected by WB and validated by Sanger sequencing. The sequences of sgRNA and primers for amplification of the sgRNA-targeted sequence of the *p190A* gene are listed in Supplementary Tables 3 and 4.

### RhoA activity assays

For quantitative analysis of active RhoA-GTP levels, the Rho Pull-down Activation Assay (Cytoskeleton, USA) was used. Briefly, Ishikawa cells were harvested in cell lysis buffer (supplied by the manufacturer). After measurement of the protein concentration with Precision Red, equal amounts of cell lysates were incubated with rhotekin-RBD beads. The amount of bound active RhoA was detected by western blotting with anti-RhoA antibody.

### Luciferase reporter assays

293T cells were transfected with the luciferase reporter SRE-Luc, green fluorescent protein (GFP) normalization plasmid, and p190A wild-type or mutant plasmid. Cells were cultured in serum-free medium for 24 h, and GFP intensity was measured using a fluorometer. The cells were lysed, and luciferase activities were detected using a luciferase assay kit from Promega. Luciferase activity was normalized against GFP intensity and p190A protein expression.

### RNA isolation, RNA sequencing, and sequencing data analysis

Total RNA was isolated from Ishikawa cells by TRIzol reagent (Tiangen, China). The amount and quality of the total RNA samples were determined by using a NanoDrop 2000 spectrophotometer (Thermo, USA). In addition, the RNA integrity was evaluated by an Agilent 2100 Bioanalyzer (RNA integrity number > 7) using the RNA Nano 6000 Assay Kit (Agilent Technologies, USA). mRNA was purified by a poly oligo-d (T) probe and then fragmented to 200 bp. cDNA was produced, end-repair was performed after purification by the QIAquick PCR kit (Qiagen, Germany), and the adaptors were ligated. The 300 nt fragments were isolated using gel purification (2% Tris-acetate-EDTA). An mRNA library was constructed, and 18–30 nt RNA was obtained from the total RNA. The cDNA was ligated and reverse transcribed. Finally, the resulting cDNA library was sequenced by using the HiSeq 2500 platform.

All raw RNA-Seq data for each sample were initially processed by FastQC to trim the adaptor and discard low-quality reads. The remaining reads were aligned to the human genome using Tophat. Fragments per kilobase per million values, estimates of the gene expression levels, were calculated by Cufflinks. Differentially expressed genes were identified by Cuffdiff according to the following criteria: fold change > 2 and false discovery rate < 0.05.

### Immunofluorescence analysis

Ishikawa cells were plated on chamber slides and fixed with 4% paraformaldehyde at 20 °C for 30 min. Cells were permeabilized with 0.1% Triton X-100 in PBS for 15 min at 20 °C. The cells were then washed with Phosphate Buffered Saline with Tween (PBST), blocked with 5% donkey serum in PBS for 1 h, and incubated with primary antibodies in PBS at 4 °C for 12 h in the dark. After washing with PBST, fluorescence-labeled secondary antibodies were used and cells were counterstained with DAPI for 20 °C for 10 min in the dark. The slides were mounted in ProLong Gold (Thermo, USA). Cells were visualized and imaged using a confocal microscope. The antibodies used for IF analysis are listed in Supplementary Table 5.

### Samples from endometrial cancer patients

The protection of the human subject board of the hospital approved the experimental protocols, and informed consent was obtained from each patient. Paraffin-embedded tissues were obtained from 553 endometrial cancer patients (human curettage specimens, *n* = 33; and primary tumors, *n* = 520) between December 2007 and December 2018.

### P190A mutation detection and IHC analysis of specimens from endometrial cancer patients

Genomic DNA was isolated using the GeneRead DNA FFPE kit (Qiagen, Germany) from the tumor-rich regions (at least 50% of tumor cells) of the FFPE samples assessed by two independent pathologists. In the samples with mutations, the nontumor mucosa was also extracted from the same slide and underwent the same approach as that for validating the somatic mutation. Supplementary Table 4 lists the 24 pairs of primer sets covering the CDS of p190A. PCR amplifications were performed using 2× Taq Master Mix (Vazyme Biotech, China). PCR products were purified using the QIAquick Gel Extraction Kit (Qiagen, Germany) following the manufacturer’s instructions and used for Sanger sequencing. Mutations in POLE (exon 9, exon 13, and exon 14) were also detected by Sanger sequencing. IHC analysis methods were described previously.^27^ The antibodies used for IHC analysis are listed in Supplementary Table 5.

### Statistical analysis

Data are shown as the mean ± SD for the experiments repeated at least three replicates. Differences between two groups were analyzed using the paired Student’s *t*-test unless otherwise specified; *p < 0.05 **p < 0.01 and ***p < 0.001.

## Supplementary information

Supplementary Materials

Supplementary Tables

## Data Availability

Detailed information on the reagents and sequences of primers, sgRNAs and shRNAs can be found in Supplementary Tables 3–5. RNA-seq data are presented in Supplementary Table 1. The clinical information of tumor specimens can be found in Supplementary Table 2. For original data, please contact kungao@tongji.edu.cn.
