# Supplementary material for: p190A inactivating mutations cause aberrant RhoA activation and promote malignant transformation via the Hippo-YAP pathway in endometrial cancer
Source: Signal Transduct Target Ther. 2020 May 27;5:81. doi: 10.1038/s41392-020-0170-6 (PMC7250911; doi:10.1038/s41392-020-0170-6)
Supplement: Supplementary file 1 — Supplementary Materials [file 41392_2020_170_MOESM1_ESM.docx]

Supplementary Materials for

p190A inactivating mutations cause aberrant RhoA activation and promote malignant transformation via the Hippo-YAP pathway in endometrial cancer

Xiaoli Wen, Jing Wan, Qizhi He, Mengfei Wang, Shuangdi Li, Mei Jiang, Zhen Qian, Binya Liu, Wen Lu, Kai Wang, Kun Gao and Xiaoping Wan.

Correspondence to: kungao@tongji.edu.cn; wanxiaoping61@126.com

**This PDF file includes:**

Figures. S1 to S8

**Other Supplementary Materials for this manuscript include the following:**

Data S1 to S5

**Supplementary Table. 1** The differentially expressed genes in p190A KO Ishikawa cells

**Supplementary Table. 2** POLE/p190A mutation status, IHC scores in 23 cases of endometrial cancer specimens and the associated clinical information

**Supplementary Table. 3** shRNA/sgRNA sequence information

**Supplementary Table. 4** Primer sequence information

**Supplementary Table. 5** Antibody information

**
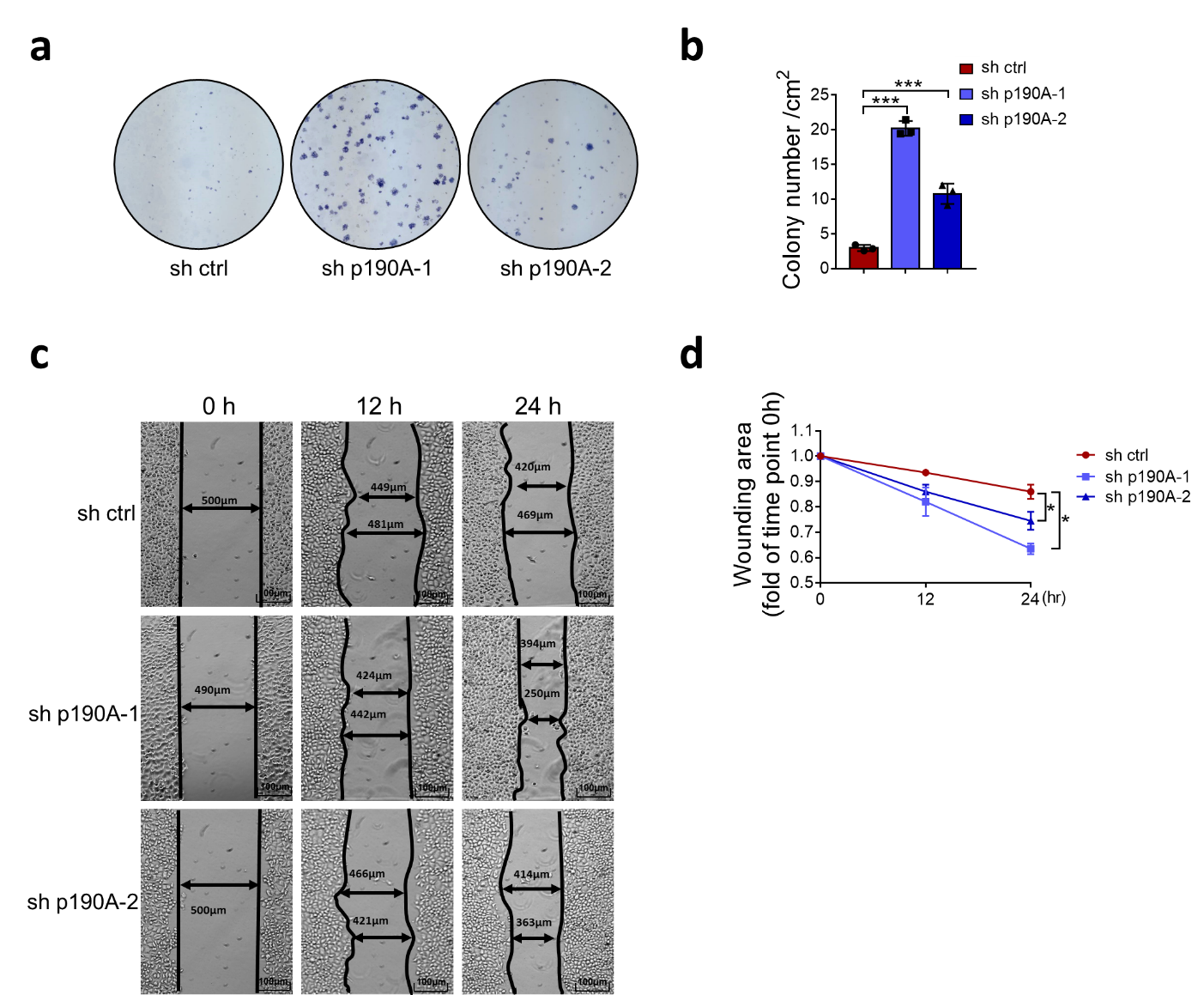
**

Figure. S1.

**P190A knockdown promotes cell proliferation and migration in Ishikawa cells** (related to Figure. 2).

(a, b) The colony formation analysis of Ishikawa cells stably expressing sh ctrl, sh p190A-1, or sh p190A-2 (a), and the quantitative data are shown in (b). Data are shown as means ± SD (n=3).

(c, d) Scratch-wounding analysis of Ishikawa cells stably expressing sh ctrl, sh p190A-1, or sh p190A-2 (c), and the quantitative data are shown in (d). Data are shown as means ± SD (n=3). Scale bar, 100 μm

**
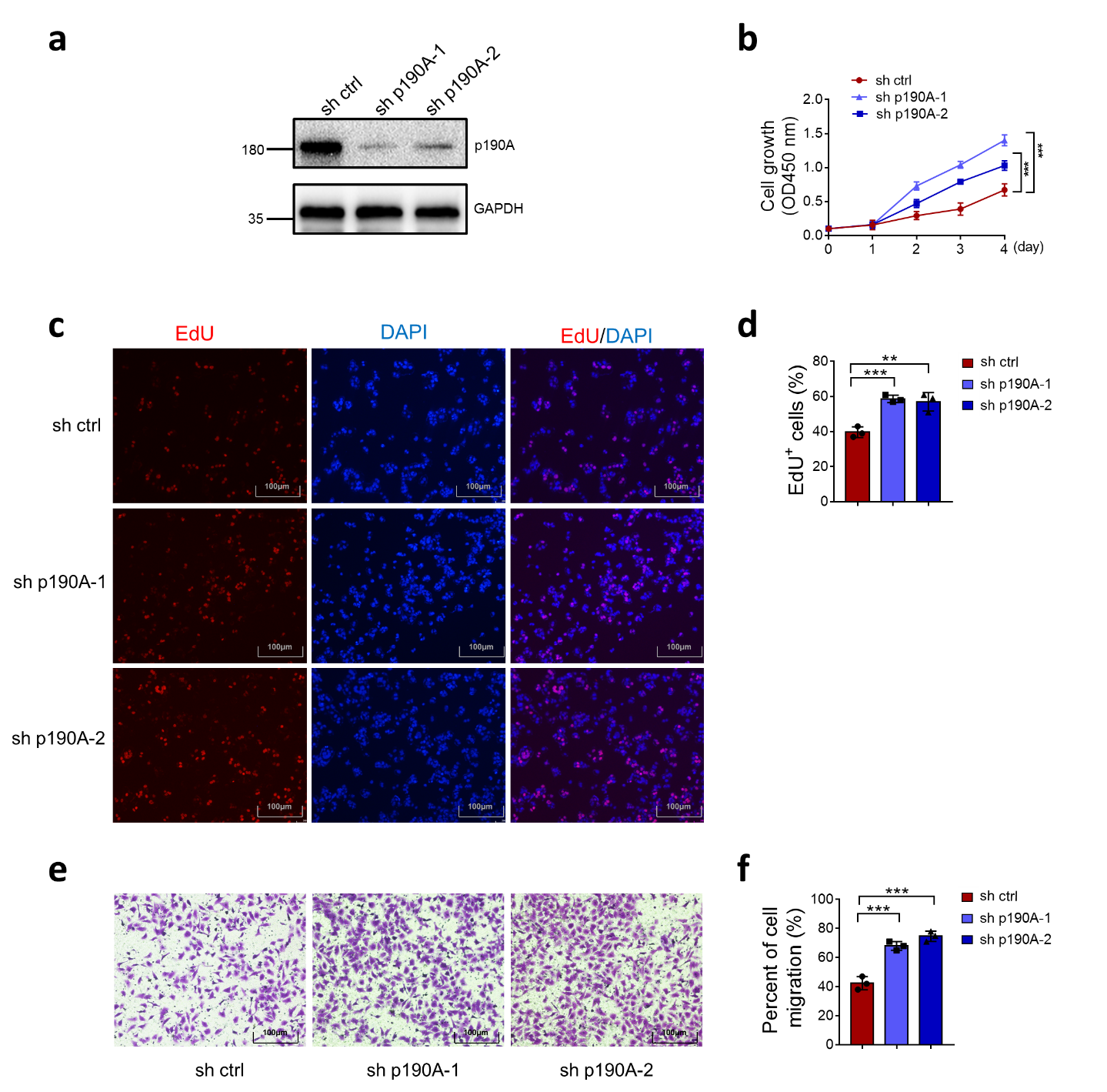
**

Figure. S2.

**P190A knockdown promotes proliferation and migration in KLE cells.**

(a) Western blot of indicated proteins in WCLs from KLE cells stably expressing sh ctrl, sh p190A-1, or sh p190A-2.

(b) CCK-8 cell proliferation analysis of KLE cells stably expressing sh ctrl, sh p190A-1, or sh p190A-2. Data are shown as means ± SD (n=3).

(c, d) The EdU incorporation analysis of Ishikawa cells stably expressing sh ctrl, sh p190A-1, or sh p190A-2 (c), and the quantitative data are shown in (d). Data are shown as means ± SD (n=3). Scale bar, 100 μm.

(e, f) Transwell incorporation analysis of KLE cells stably expressing sh ctrl, sh p190A-1, or sh p190A-2 (e), and the quantitative data are shown in (f). Data are shown as means ± SD (n=3). Scale bar, 100 μm.


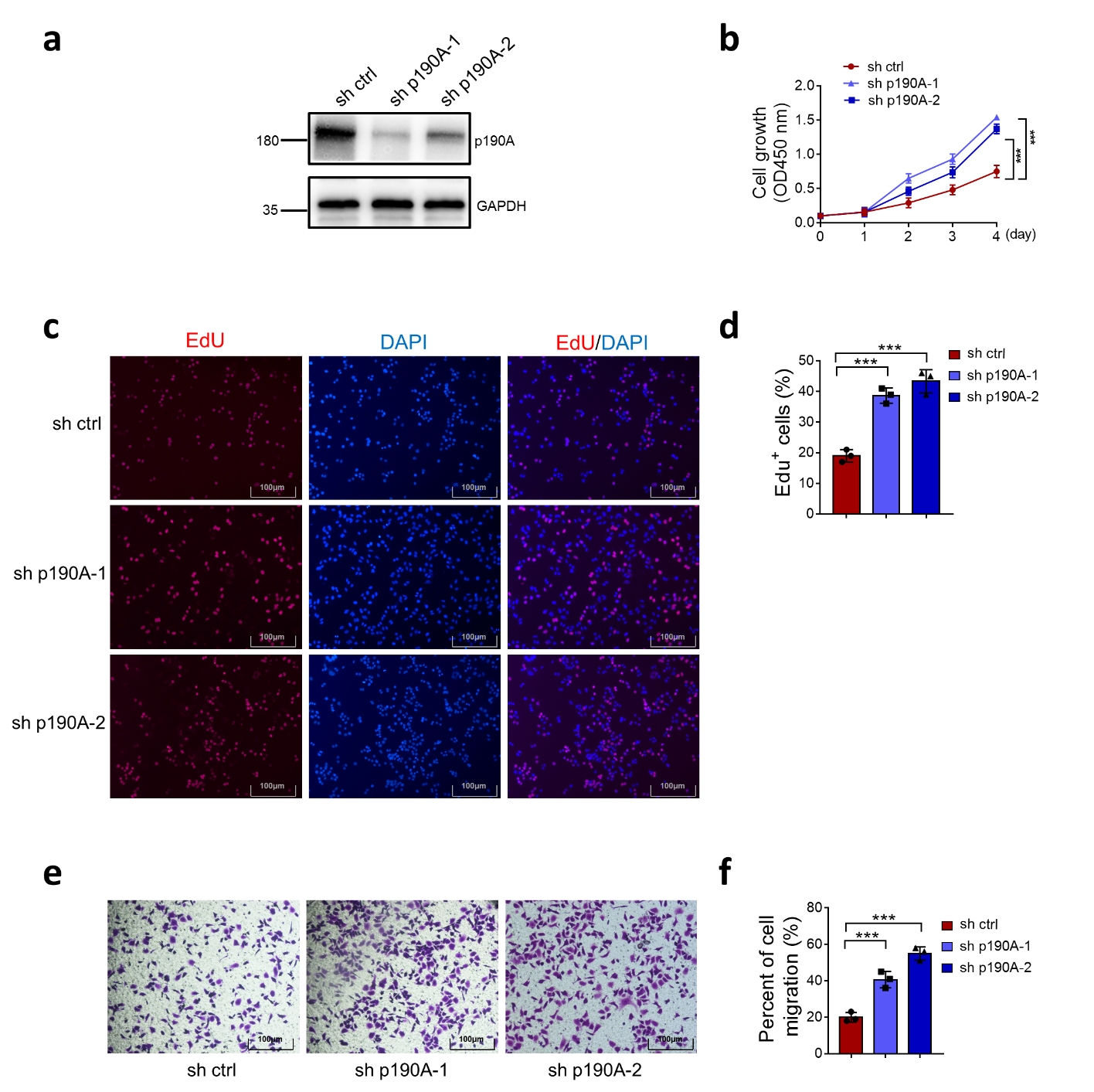


Figure. S3.

**P190A knockdown promotes proliferation and migration in RL95-2 cells.**

(a) Western blot of indicated proteins in WCLs from RL95-2 cells stably expressing sh ctrl, sh p190A-1, or sh p190A-2.

(b) CCK-8 cell proliferation analysis of RL95-2 cells stably expressing sh ctrl, sh p190A-1, or sh p190A-2. Data are shown as means ± SD (n=3).

(c, d) The EdU incorporation analysis of RL95-2 cells stably expressing sh ctrl, sh p190A-1, or sh p190A-2 (c), and the quantitative data are shown in (d). Data are shown as means ± SD (n=3). Scale bar, 100 μm.

(e, f) Transwell incorporation analysis of RL95-2 cells stably expressing sh ctrl, sh p190A-1, or sh p190A-2 (e), and the quantitative data are shown in (f). Data are shown as means ± SD (n=3). Scale bar, 100 μm.

**
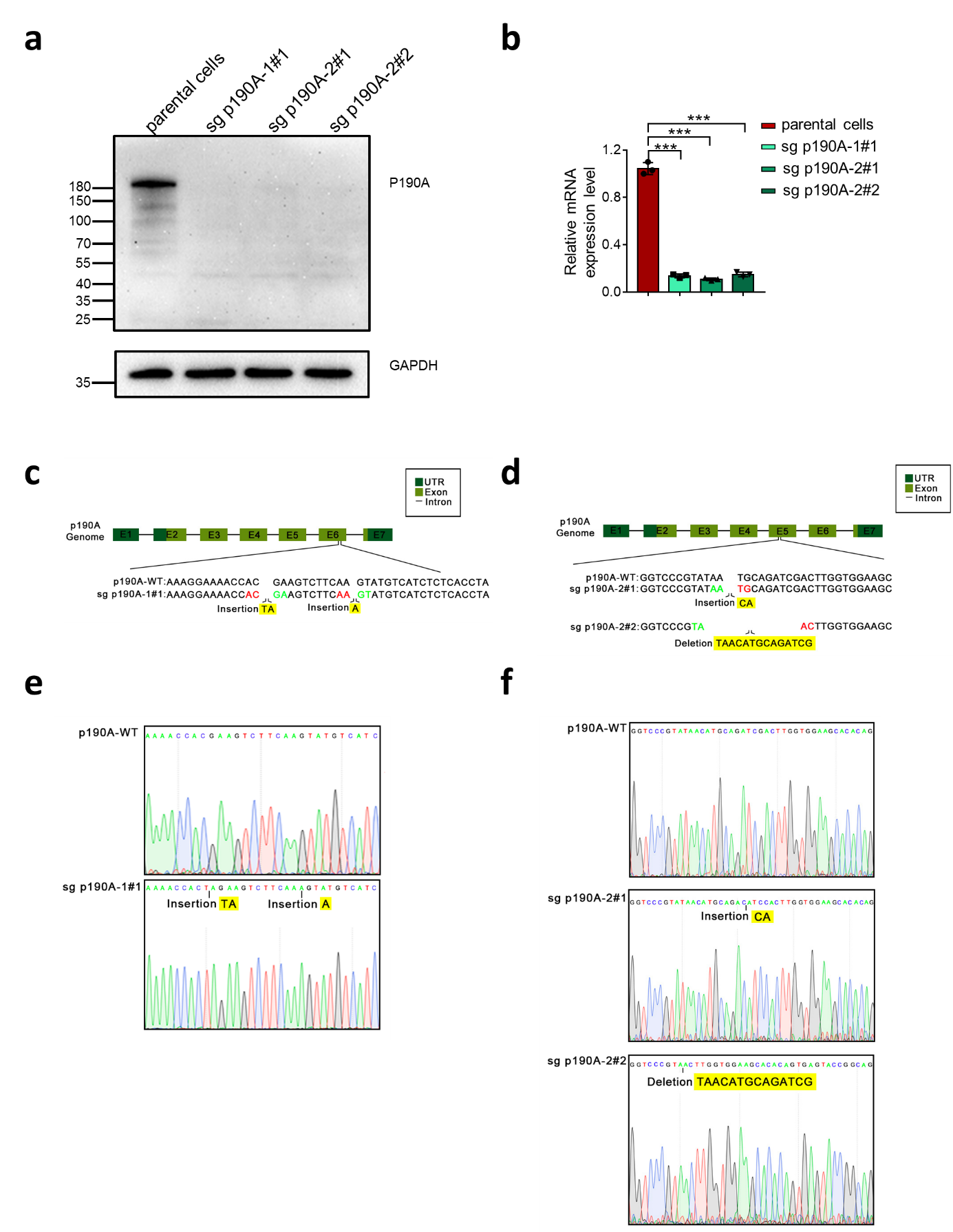
**

Figure S4.

**Validation of p190A knockout in Ishikawa cells**.

(a) Western blot of indicated proteins in WCLs from Ishikawa cells with p190A KO by CRISPR-Cas9 methods. Parental Ishikawa cells were used as a control.

(b) RT-qPCR assessment of p190A mRNA expression in parental and p190A-KO Ishikawa cells. The mRNA level of GAPDH was used for normalization. Data are shown as means ± SD (n=3). ***p<0.001.

(c) Schematic of CRISPR/Cas9-mediated knockout of p190A by sgRNA-1 in Ishikawa cells.

(d) Schematic of CRISPR/Cas9-mediated knockout of p190A by sgRNA-2 in Ishikawa cells. Two clones were showed.

(e) Sanger sequencing confirming that the p190A gene was edited by sgRNA-1 in p190A-KO Ishikawa cells.

(f) Sanger sequencing confirming that the p190A gene was edited by sgRNA-2 in p190A-KO Ishikawa cells. Two clones were showed.


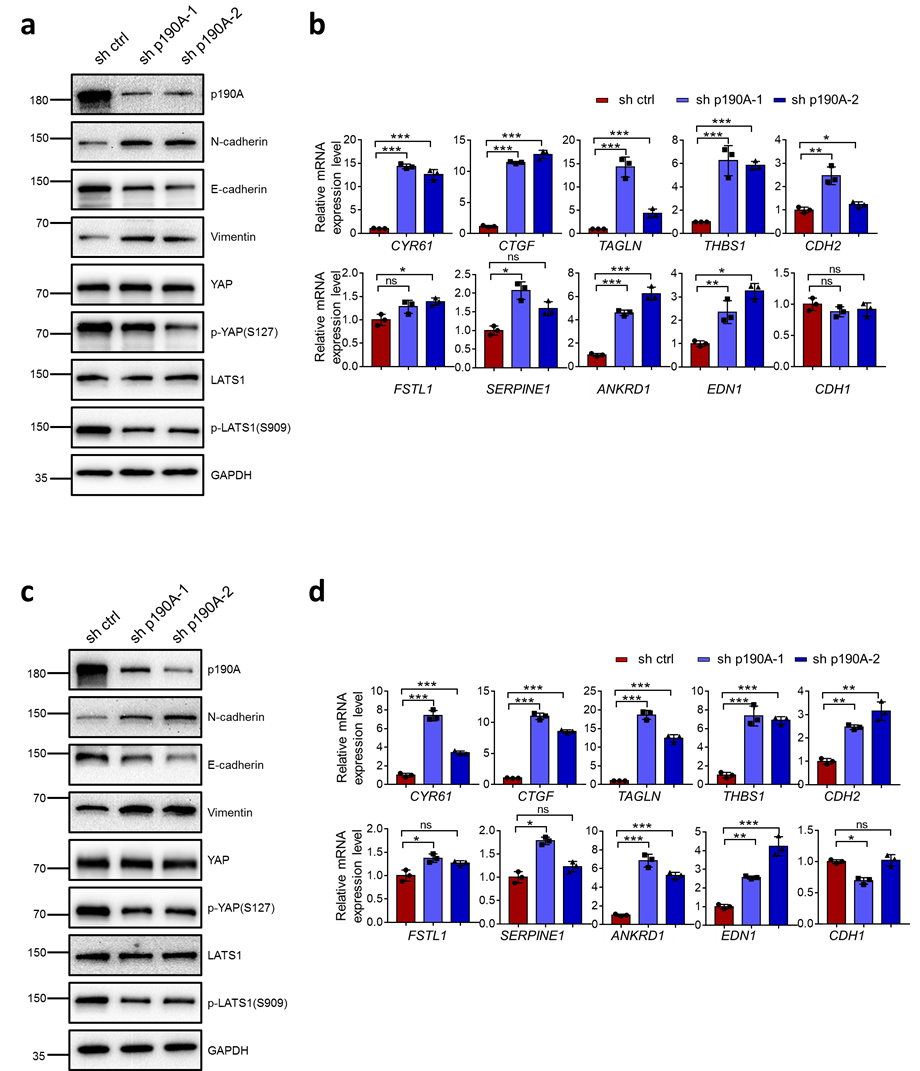


Figure S5.

**The depletion of p190A in KLE or RL95-2 cells leads to EMT and aberrant Hippo-YAP pathway activity**.

(a) Western blot of indicated proteins in WCLs from KLE cells stably expressing sh ctrl, sh p190A-1, or sh p190A-2.

(b) RT-qPCR measurement of the mRNA expression of EMT-related genes in KLE cells stably expressing sh ctrl, sh p190A-1, or sh p190A-2. Data are shown as means ± SD (n=3).

(c) Western blot of indicated proteins in WCLs from RL95-2 cells stably expressing sh ctrl, sh p190A-1, or sh p190A-2.

(d) RT-qPCR measurement of the mRNA expression of EMT-related genes in RL95-2 cells stably expressing sh ctrl, sh p190A-1, or sh p190A-2. Data are shown as means ± SD (n=3).

**
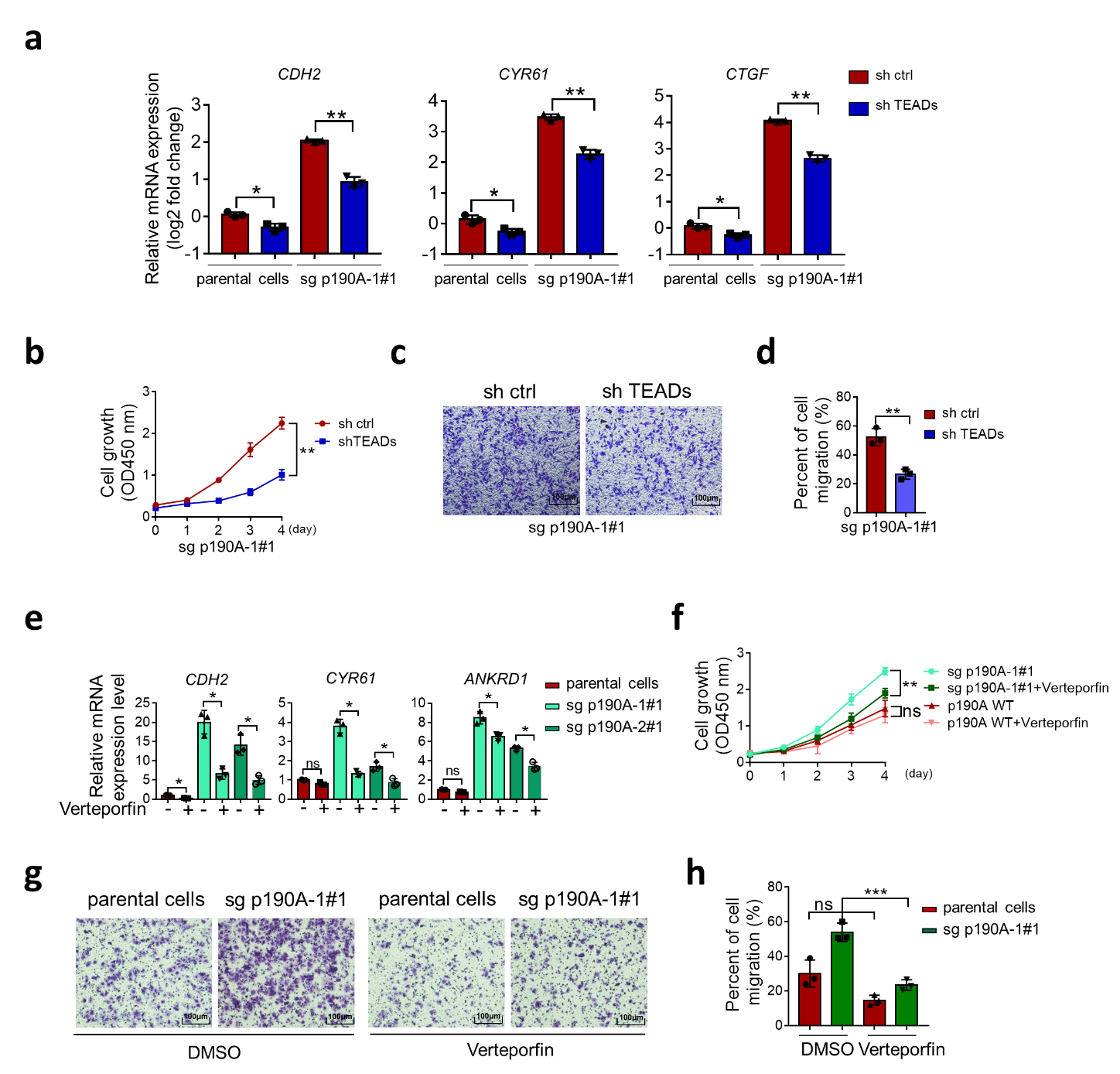
**

Figure S6.

**KO of p190A in Ishikawa cells leads to aberrant Hippo-YAP pathway activity** (related to Figure 4).

(a) RT-qPCR measurement of the mRNA expression of EMT-related genes in parental and p190A KO Ishikawa cells stably expressing sh ctrl or sh TEADs. Data are shown as means ± SD (n=3).

(b) CCK-8 cell proliferation analysis of p190A KO Ishikawa cells stably expressing sh ctrl or sh TEADs. Data are shown as means ± SD (n=3).

(c, d) Transwell migration analysis of p190A KO Ishikawa cells stably expressing sh ctrl or sh TEADs (c), and the quantitative data are shown in (d). Data are shown as means ± SD (n=3). Scale bar, 100 μm.

(e) RT-qPCR measurement of the mRNA expression of EMT-related genes in parental and p190A KO Ishikawa cells treated with DMSO or verteporfin (2 μM) for 48h. Data are shown as means ± SD (n=3).

(f) CCK-8 cell proliferation analysis of parental and p190A KO Ishikawa cells treated with DMSO or verteporfin (2 μM) for indicated times. Data are shown as means ± SD (n=3).

(g, h) Transwell migration analysis of parental and p190A KO Ishikawa cells treated with DMSO or verteporfin (g) for 16h, and the quantitative data is shown in (h). Data are shown as means ± SD (n=3). Scale bar, 100 μm

**
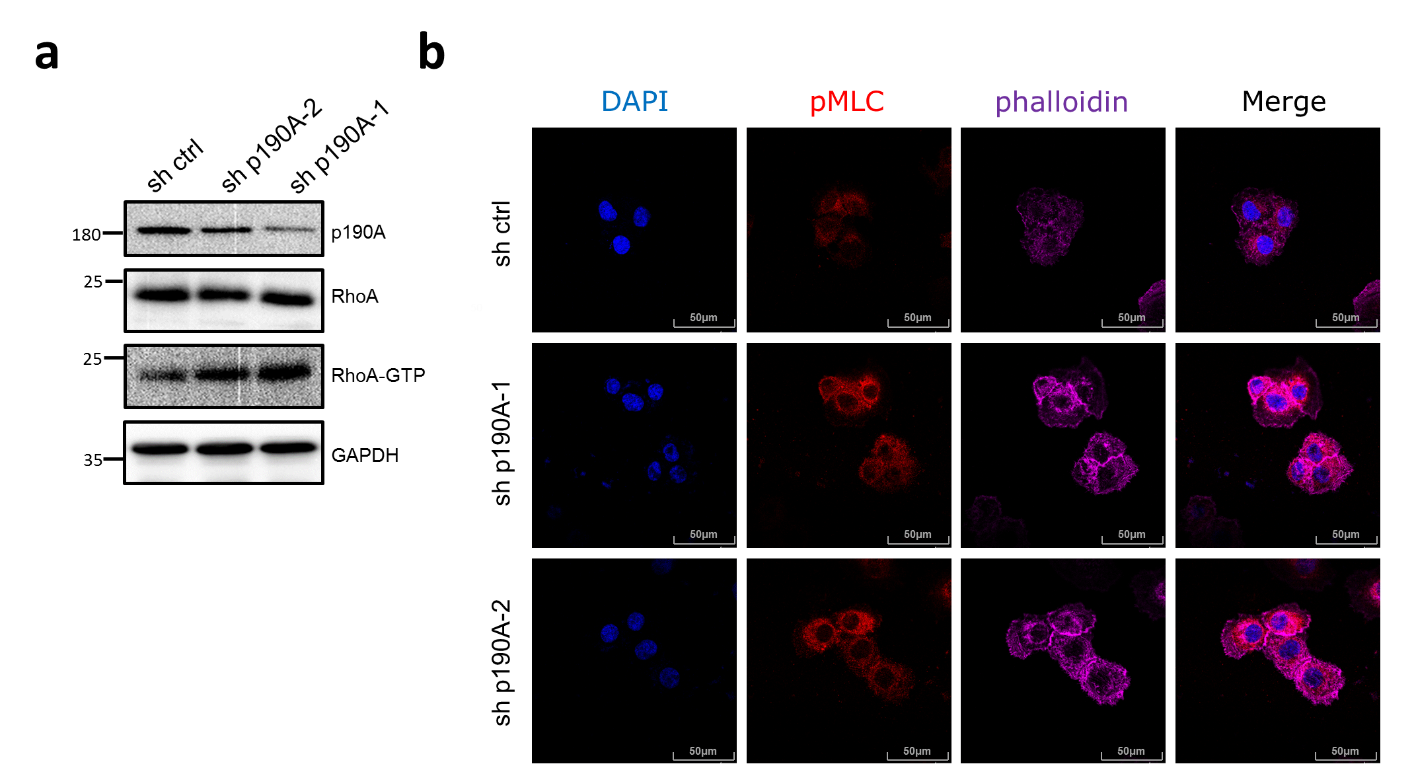
**

Figure S7.

**The knockdown of p190A in Ishikawa cells led to an increased cellular level of active RhoA.**

(a) Active RhoA protein levels were measured in Ishikawa cells stably expressing sh ctrl, sh p190A-1, or sh p190A-2 by RBD pull-down assay.

(b) Representative immunofluorescence images of Ishikawa cells stably expressing sh ctrl, sh p190A-1, or sh p190A-2 stained with phospho-MLC (pMLC), phalloidin and DAPI. Scale bar, 50μm.

**
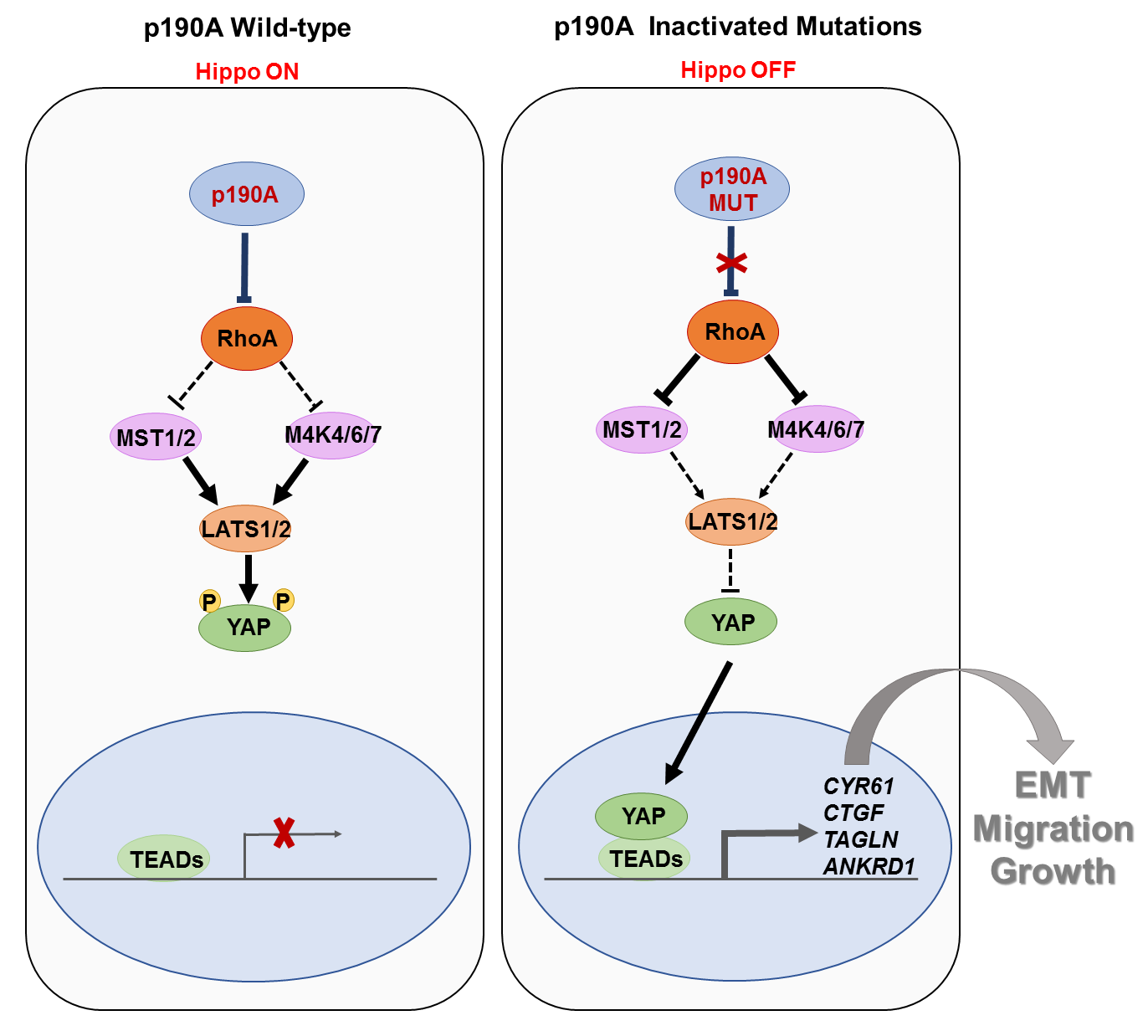
**

Figure S8.

**Schematic of the proposed mechanism through which p190A mutations enhance endometrial tumorigenesis via the Hippo-YAP pathway.**
